# Accuracy of gestational age estimation from last menstrual period among women seeking abortion in South Africa, with a view to task sharing: a mixed methods study

**DOI:** 10.1186/s12978-017-0365-7

**Published:** 2017-08-22

**Authors:** Deborah Constant, Jane Harries, Jennifer Moodley, Landon Myer

**Affiliations:** 1 Women’s Health Research Unit, School of Public Health and Family Medicine, Faculty of Health Sciences, University of Cape Town, Cape Town, South Africa; 2 Division of Epidemiology and Biostatistics, School of Public Health and Family Medicine, Faculty of Health Sciences, University of Cape Town, Cape Town, South Africa

**Keywords:** Last menstrual period, Medical abortion, Eligibility, task sharing

## Abstract

**Background:**

The requirement for ultrasound to establish gestational age among women seeking abortion can be a barrier to access. Last menstrual period dating without clinical examination should be a reasonable alternative among selected women, and if reliable, can be task-shared with non-clinicians. This study determines the accuracy of gestational age estimation using last menstrual period (LMP) assessed by community health care workers (CHWs), and explores providers’ and CHWs’ perspectives on task sharing this activity. The study purpose is to expand access to early medical abortion services.

**Methods:**

We conducted a multi-center cross-sectional study at four urban non-governmental reproductive health clinics in South Africa. CHWs interviewed women seeking abortion, recorded their LMP and gestational age from a pregnancy wheel if within 63 days. Thereafter, providers performed a standard examination including ultrasound to determine gestational age. Lastly, investigators calculated gestational age for all LMP dates recorded by CHWs. We compared mean gestational age from LMP dates to mean gestational age by ultrasound using t-tests and calculated proportions for those incorrectly assessed as eligible for medical abortion from LMP. In addition, in-depth interviews were conducted with six providers and seven CHWs.

**Results:**

Mean gestational age was 5 days (by pregnancy wheel) and 9 days (by LMP calculation) less than ultrasound gestational age. Twelve percent of women were eligible for medical abortion by LMP calculation but ineligible by ultrasound. Uncertainty of LMP date was associated with incorrect assessment of gestational age eligibility for medical abortion (*p* = 0.015). For women certain their LMP date was within 56 days, 3% had ultrasound gestational ages >70 days. In general, providers and CHWs were in favour of task sharing screening and referral for abortion, but were doubtful that women reported accurate LMP dates. Different perspectives emerged on how to implement task sharing gestational age eligibility for medical abortion.

**Conclusions:**

If LMP recall is within 56 days, most women will be eligible for early medical abortion and LMP can substitute for ultrasound dating. Task sharing gestational age estimation is feasible in South Africa, but its implementation should meet women’s privacy needs and address healthcare workers’ concerns on managing any procedural risk.

## Plain English summary

Medical abortion was introduced in 2011 in South Africa, is very effective and safe for abortion up to 9 weeks and does not require specialized surgical skills. However, introductory protocols required an ultrasound examination be done to date the pregnancy using equipment and expertise not widely available, especially in more remote rural areas. If women can remember the first day of their last menstrual period (LMP), this can be used to work out their pregnancy duration, and if community health workers (CHWs) can ascertain pregnancy duration from women’s LMP, this could assist expansion of medical abortion services into primary care public health facilities across the country.

This study identified women for whom LMP dating would suffice to ensure a safe and effective medical abortion, and gauged nurses’ and CHWs’ opinions on how they could share responsibility for this activity.

CHWs recorded LMP among women seeking abortion at reproductive health clinics in South Africa, and pregnancy duration was calculated. Thereafter, providers performed an ultrasound examination. Proportions were calculated for women incorrectly assessed as eligible for medical abortion from LMP overall, and according to different LMP cut-off dates. Providers and CHWs were also interviewed.

A small percentage (3%) of women underestimated their LMP such that the chances of continuing a pregnancy may increase and women may experience more side effects. In general, providers and CHWs favored sharing responsibility for establishing gestational age eligibility for medical abortion, but were doubtful that women reported accurate LMP dates. They also had different perspectives on ways to implement this. Future implementation of task sharing gestational age estimation for medical abortion should meet women’s privacy needs as well as health-care workers’ concerns about possible health risks.

## Background

Abortion on request in the first trimester of pregnancy was made legal in South Africa in 1997, and mifepristone for medical abortion was approved for use in medical abortion by the South African drug regulatory authority in 2001. The combined regimen of mifepristone and misoprostol has been available in the private sector since 2001 and in the public sector, where the majority of women access healthcare, since 2010 [1]. Medical abortion for women with pregnancies up to 9 weeks' gestational age has gradually been introduced into primary care public sector health facilities around the country, however expansion of the service has been slow, in part due to a scarcity of trained providers, and rural districts are generally underserved [2]. Access has been limited to facilities or referral centers that have ultrasound equipment and the requisite expertise as the 2010 provincial medical abortion guidelines [1] mandated the use of ultrasound to determine gestational age eligibility for all abortion procedures – in contrast to other more recent recommendations where routine use of pre-abortion ultrasound is not an absolute requirement [3] and may be substituted with bimanual clinical examination [4].

Medical abortion involves taking pills, and the requirement for an ultrasound assessment of gestational age can create a significant barrier since providing the medication and the information to safely and effectively take it is simple and well documented. Simplifying gestational age assessment could have a major impact on access and service delivery in a range of contexts, particularly at primary care level. If gestational age based on last menstrual period (LMP) can be accurately assessed by community health care workers (CHWs) in supportive roles, this could potentially reduce delays though appropriate referral to facilities, improve access and save women’s and providers’ time. In addition, this approach could save healthcare resources in other ways such as not buying unnecessary equipment or requiring services of a trained sonographer.

The requirement for an ultrasound examination is a barrier to abortion access in many resource-limited settings and researchers have long challenged this prerequisite [5–7]. More recently the accuracy of LMP-based gestational age has again been under review [8] with efforts to identify criteria that would ensure a safe and effective medical abortion in terms of a woman’s gestational age, using LMP rather than ultrasound [9]. An earlier study from South Africa found that LMP-based gestational age was accurate on average, compared to ultrasound dating, but that there were high levels of uncertainty among 12% of women [6]. Other published studies demonstrated better accuracy of LMP-based gestational age estimations [9], suggesting that LMP recall may vary according to country context [5, 7, 9, 10]. It is also evident that LMP recall is more difficult for women with irregular menstrual cycles [11] and that recall can be significantly biased among women seeking abortion when the pregnancy is advanced [12]. Calculation of gestational age from LMP to the current date has been aided by the use mechanical pregnancy wheels (2 concentric discs with dials on their perimeters, the upper one able to rotate), however electronic calculators have been shown to be more accurate as they involve less user-error [13, 14].

To expand access to medical abortion where barriers exist due to the ultrasound requirement and provider shortages, the most recent WHO guidelines [15] recommend task-shifting LMP-based gestational age estimation to lay health workers or for women to evaluate this themselves. The research evidence guiding these recommendations suggests task sharing this component of care could be safe and feasible for abortion in the first trimester where services are often offered in primary care settings, but cautions that further rigorous contextual research is needed in this regard. Similar recommendations were made with respect to self-assessment of gestational age for medical abortion eligibility by women themselves [15].

This study analyzes data from a larger multi-country validation study of a medical abortion eligibility checklist tool for use by CHWs as a means to improve access to medical abortion though expansion of the scope of the CHWs' roles [16]. In this sub-study we present evidence on the effectiveness of CHWs' assessment of gestational age among women seeking abortion and provider opinion on task sharing this activity. We compare three methods of gestational age estimation: 1) LMP plus digital calculation by investigators of intervening days for gestational age estimation, 2) LMP plus pregnancy wheel used by CHWs for gestational age estimation, and 3) gestational age from ultrasound examination by clinicians. In addition, we describe providers’ and CHWs’ perspectives on task sharing eligibility assessment for medical abortion. The purpose of the study is to advance the potential for gestational age eligibility for medical abortion to be performed by CHWs or by women themselves, without the need for ultrasound examination, in order to expand access to medical abortion services in South Africa.

## Methods

### Study participants and setting

Women were recruited between August and October, 2012 in urban sexual and reproductive health clinics providing abortion in Kwazulu-Natal (three clinics) and the Western Cape (one clinic). The sample size calculated for the parent study was based on the assumption that 60% of women seeking abortion would be eligible for medical abortion. To achieve a two-sided 90% confidence interval for 60% ±15% sensitivity and 80% ±15% specificity, it was estimated a sample size of 211 was needed [16]. The project was conducted at non-governmental organization (NGO) clinics with providers (nurse clinicians) trained in medical abortion as well as certified CHWs in counselling roles; at the time no public sector service was able to meet these requirements. For this study, CHWs received 2 days of didactic training on the menstrual cycle, abortion methods and eligibility, and use of the pregnancy wheel.

### Study procedures

#### Gestational age assessments

Women seeking abortion at study clinics were approached by a research assistant to determine interest and eligibility. Eligibility criteria were: 18 years or older, able to speak local languages, and willing and able to give written informed consent. Eligible women provided written consent, completed a socio-demographic questionnaire with the research assistant, and were interviewed by CHWs using the medical abortion assessment toolkit developed for the parent study. CHWs recorded known or estimated LMP with the aid of a calendar when needed, used the pregnancy wheel to establish eligibility for medical abortion based on gestational age, and recorded the gestational age from the wheel if within 63 days. Thereafter women were seen by a provider who completed a standard clinical examination protocol including an ultrasound examination for eligibility for medical abortion and recorded these data. None of the women had been informed of their gestational age prior to joining the study, and the CHWs were masked as they interviewed women prior to them seeing the clinician.

#### Analysis of gestational age assessments

The main outcome was the difference between LMP-based gestational age estimates and ultrasound-based gestational age. Two methods of LMP-based gestational age were included in this analysis; firstly, from CHWs' estimate of gestational age using the pregnancy wheel (only recorded where wheel gestational age was within 63 days - referred to as *GA by wheel*), and secondly, by investigator post-hoc calculation of gestational age based on recorded LMP date (referred to as *GA by calculation*) for all participants. Mean LMP-based gestational age (LMP-based GA) estimations were compared to ultrasound gestational age (U/S GA) using paired t-tests and Bland Altman plots. For each of the LMP-based methods we calculated the proportion of cases in the caution zone, described as those with an LMP-based GA within 63 days, but with U/S GA beyond 63 days [5]. Conflicting classifications of cases into the caution zone using the pregnancy wheel compared to the digital calculation were identified and described. Potential associations for being in the caution zone and for being unsure of LMP date were assessed using Kruskal-Wallis and Chi-squared tests for continuous and categorical variables respectively. Following the approach in recently published literature [9], we then calculated the proportion of women whose *GA by calculation* was either <=56 or <=63 days but for whom U/S GA was beyond 63 days or 70 days. Subgroups in this analysis included women who stated they were sure of their LMP date.

#### In-depth interviews

In-depth interviews (IDIs) were done with all CHWs and providers who had assessed at least six women in the quantitative study when data collection for gestational age assessment was complete. Following informed consent, interviews were conducted by an experienced study investigator in English, according to a semi-structured interview guide. IDIs were recorded and transcribed, and stripped of personal identifiers. Transcripts were analyzed using a thematic approach based on the interview guide.

All participants in the study provided written informed consent and their confidentiality was safeguarded. Women received ZAR50 reimbursement on conclusion of their participation in the study for any expenses incurred; providers and CHWs were not reimbursed for participating in the IDIs. The study protocol was approved by the World Health Organization Research Ethics Review Committee and the University of Cape Town’s Human Research Ethics Committee.

## Results

### Gestational age assessments

**Fig. 1:**
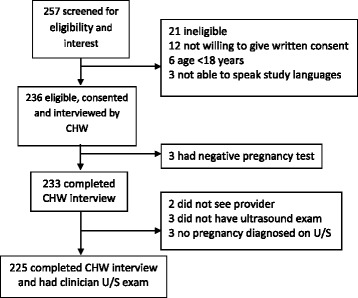
Flow chart of participants enrolled into the study

Between August and October 2012, 236 women were enrolled. Of these, 11 were excluded from this analysis (Fig. 1) leaving 225 pairs with complete data; three had a negative pregnancy test according to the CHW, two did not see the clinician, three did not have pregnancies confirmed by ultrasound, and three did not have an ultrasound examination due to disruptions in clinic routines.

There were 94 participants from the Western Cape and 131 from KwaZulu-Natal (Table 1). Significant differences in demographic or reproductive characteristics between the two regions included a higher percentage of women from the Western Cape with prior abortions, with paid work, who used a contraceptive method in the past year and who were not sure of their LMP as recorded by the CHW.

**Table 1:** Socio-demographic and reproductive characteristics of participants

	Western Cape (*n* = 94)	Kwazulu/Natal (*n* = 131)	Total (*n* = 225)	p-value^a^
Age, (years)				
Median (IQR)	26 (23.0-31.3)	26 (23.2-29.3)	26.0 (23.0-30.0)	0.452
School education, (years)				
Completed school education (Grade 12) (Yes) n (%)	66 (70.2)	92 (70.2)	158 (70.2)	1.000
Paid employment (Yes) n (%)	63 (67.0)	31 (33.0)	94 (40.9)	<0.001
Previous pregnancies, n (%)				0.868
0	20 (21.3)	31 (23.7)	51 (22.7)	
1	30 (31.9)	43(32.8)	73 (32.4)	
2+	44 (46.8)	57 (43.5)	101 (44.9)	
Prior abortion (among those ever pregnant) n (%)	21/74 (27.3)	10/100 (10.0)	31/17 (17.2)	0.003
Used contraception in last year (Yes) n (%)	78 (83.0)	60 (45.8)	138 (61.3)	<0.001
Sure of LMP date (Yes) n (%)	73 (77.7)	121 (92.4)	194/225 (86.2)	0.002

^a^ Chi-squared tests for comparing categories, Kruskal-Wallis test for comparing medians

CHWs established known or estimated LMP date for all 225 participants. Using the pregnancy wheel, gestational age was within 63 days and was recorded by the CHW for 170/225 (76%), and gestational age from LMP calculated for all 225 participants, which included those with later gestations. Mean LMP-based GA *by wheel* for the subset of 170 participants (47 days, SD = 9.3) was 9 days shorter than U/S GA (56 days, SD = 17.2; *p* < 0.001) and for all participants, mean LMP-based GA *by calculation* (56 days, SD = 17.7 days) was 5 days shorter than U/S GA (61 days, SD = 21.3; *p* < 0.001).

**Fig. 2:**
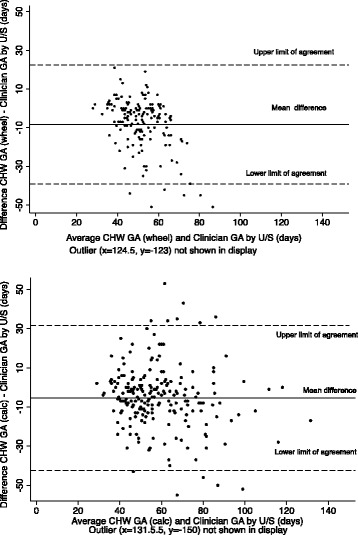
Bland Altman plots comparing LMP-based GA with U/S GA

Bland-Altman plots illustrating the difference between LMP GA and U/S GA against average gestational age are shown in Fig. 2. The limits of agreement (GA *by wheel* = −39 to 22 days; GA *by calculation* = −42 to 31 days) were wide for both methods. The plots demonstrate increasing underestimation using LMP-based GA as the pregnancy progresses, which was more prominent for GA *by wheel* than for GA *by calculation*.

LMP GA *by wheel* allocated 31/170 (18%) and GA *by calculation* allocated 28/225 (12%) to the caution zone. Where there was conflicting classification into the caution zone by GA *by wheel* compared to GA *by calculation*, four cases were underestimated by 3 to 6 days and one case by 30 days using the wheel compared to the calculation. In contrast, two cases were overestimated using the wheel and classified as ineligible so not in the caution zone, but were eligible according to calculation from LMP. Further analysis of the caution zone using the LMP GA *by calculation* results showed that most (22/28; 79%) had gestational ages between 9 and 12 weeks. Of the six cases with a gestational age > 12 weeks, three were unsure of their LMP, all had prior pregnancies, and none spoke English as their home language (English home language was uncommon among all participants; 31/225, 13.8%).

The only significant association with being in the caution zone was uncertainty of LMP date. Three participants did not know their LMP date, while 28 were unsure of this date. Combining “not sure” and “don’t know”, the association with being in the caution zone was significant for GA *by wheel* (*p* < 0.001), and for GA *by calculation* (*p* = 0.015). By definition, women with later U/S GA were more likely to be in the caution zone. No significant associations with being in the caution zone were found for age, home language, employment status, marital status, numbers of previous pregnancies or any previous abortions, use of any contraceptive method in the past year, use of an injectable contraceptive in the last year; nor were there any significant associations by province, community health worker or by clinician. There were no significant associations with being unsure of LMP other than increasing gestational age (*p* = 0.035).

Table 2 shows proportions of participants with U/S GA beyond 63 days according to two cut-offs of LMP-based GA: Those with LMP <=56 days prior and those with LMP <=63 days prior. A sub-analysis is included for participants who stated they were sure of their LMP.

**Table 2:** Gestational ages for subgroups of women by LMP and by Ultrasound

Gestational age	All participants	Participants “sure” of LMP
N with LMP and U/S GA recorded	225	194
n (%) GA ≤ 63 days by U/S	152/225 (67.6)	140/194 (72.2)
N with GA ≤ 63 days by LMP	159	143
n (%) GA ≤ 63 days by U/S	131/159 (82.4)	123/143 (86.0)
N with GA ≤ 56 days by LMP	128	118
n (%) GA ≤ 63 days by U/S	115/128 (89.8)	110/118 (93.2)

From these findings, of the women reporting their LMP as within 63 days, 18% would have received a medical abortion at U/S GAs >63 days, (Table 2). For those “sure” of their LMP, this would have been 14%. However, of the women reporting LMP dates as within 56 days,

10% would have received a medical abortion at U/S GAs of >63 days and for those sure of their LMP, this proportion decreased to 7%. Of this 7%, 4% had U/S GAs of <70 days, and of the remaining 3%, all had U/S GAs beyond 75 and within 84 days (data not shown).

### In depth interviews

We conducted interviews with six of the 10 providers (3 completed <6 assessments, 1 was unavailable) and seven of the eight CHWs (1 completed only 3 assessments). All four clinics were represented. On average, providers were 29 years (SD: 7 years) and CHWs were 40 years old (SD: 15 years). There was one male provider and one male CHW, all had completed their high school education. Providers had between 2 and 10 years’ experience and CHWs between 10 months and 3 years’ experience working in the health sector.

### Acceptability of task sharing medical abortion eligibility assessment

Providers were supportive of the notion of task sharing assessment of women’s eligibility for medical abortion, but saw this process as preliminary screening prior to referral to the clinician for a clinical assessment. They saw the role of CHWs as confined to counselling and providing information on the abortion procedure as they believed women did not accurately report their LMP dates. In addition, they felt problems between staff and confusion for women could arise if there were conflicts between CHW’s and clinician’s estimates of gestational age and eligibility for an abortion.

*“Ultimately a professional clinician should assess gestational age” (Centre manager, NGO).*

CHWs were generally positive about their role in assessing eligibility for medical abortion. They were in agreement with providers that women were inaccurate with their LMP dates, but had a number of suggestions for ways of helping women recalling their LMP more accurately. Some CHWs felt reassured that a clinical exam would still be performed after their assessment, particularly for women who experienced menstrual irregularity. CHWs were familiar with pregnancy tests and easily followed the questions on the study form. Some found the pregnancy wheel problematic initially, although competency improved with practice.

### Implementing task sharing of medical abortion eligibility assessment

There were discrepancies between providers and CHWs and lack of agreement on a service delivery model for implementation of task sharing. Providers expressed some concerns about lack of supervision and accountability for CHWs, and that CHWs might provide information outside their scope of practice. In contrast, CHWs were enthusiastic about expanding their skills.

Providers felt it would be most beneficial for CHWs to proactively perform eligibility assessments and provide information within the community, while preserving confidentiality, rather than at the clinic. However, CHWs felt the clinic was a better place for this due to lack of privacy in their usual encounters with community, which were either group discussions or home visits. Ideas for alternative placements for CHWs to conduct assessment and provide information included government clinics, hospitals and universities, while schools and homes were not considered advisable due to stigma around abortion and confidentiality concerns. Individual assessment and referral could be done telephonically or in a private place following a group information session.

## Discussion

This clinic-based study compared LMP-based GA to an ultrasound exam to determine the potential for screening gestational age eligibility for medical abortion by CHWs or by women themselves using LMP recall. The study found that on average, differences were slightly larger than other studies in which clinicians use LMP date to estimate gestational age among women seeking abortion [5, 6, 8]. Use of the pregnancy wheel contributed to error, with 7/31 (23%) misclassifications with respect to the caution zone using the pregnancy wheel compared to a calculation from LMP date. The pregnancy wheel has been reported elsewhere as prone to inaccuracies and use of electronic pregnancy calculators is considered superior to manual calculations in clinical settings [13, 14]. Recently, online electronic pregnancy calculators for mobile phones have become readily available, are accurate and easier for women and CHWs to use, with a pregnancy wheel for back-up and in settings where this is not feasible [17].

GA *by calculation* allocated 12% to the caution zone, which is similar to earlier studies conducted in the United States (US) and South Africa [5, 6]. Uncertainty of LMP date was the only significant association with being in the caution zone in this study. While little conclusive evidence has been published on predictors for being in the caution zone [5, 6, 12], being unemployed, primigravidity, pregnancy denial and failure to recognize pregnancy signs have been associated with inaccuracies in estimation of pregnancy duration [5, 18].

Of importance is the proportion of women who would be classified as eligible for medical abortion according to their LMP but whose U/S GA is beyond the safety and efficacy limits for medical abortion. Recent research [19–22] has demonstrated that 200 mg mifepristone combined with home-administered misoprostol is safe and effective for medical abortion up to 70 days gestational age, and may be so up to 84 days, if women are given additional misoprostol, as well as sufficient counselling and support. In this study, only 3% of women who were sure that their LMP date was at least 56 days prior would have had a medical abortion beyond the 70-day limit. This is more than the 0.6% for US women in 2011, but less than the earlier study (7.8%) of US women in 2000 [9]. Currently in South Africa, medical abortion with off-site use of misoprostol is permitted up 63 days gestational age, however extending the medical abortion gestational age limit to 70 days and task sharing gestational age eligibility decisions to CHWs or to women themselves would result in a safe medical abortion for most women who state that their LMP is within 56 days. For example, of 1000 women seeking abortion and giving an LMP date no longer than 56 days earlier: If assessed by a CHW, 970 would be managed safely and effectively and 30 might need an additional intervention such as additional misoprostol or an aspiration procedure.

Fourteen percent of women stated they were uncertain or did not know their LMP date, as compared with the US (29%) and the United Kingdom (9.5%) [8]. The association between certainty of LMP and accuracy of LMP date is be expected. For women who are unsure of their LMP, additional prompts could help women reach a closer approximation. Irregular menses and absence of a personal calendar record or any significant event (e.g. birthday) have been shown to hinder LMP recall [23].

The IDIs showed health care worker’s concerns with respect to women’s ability to accurately report their LMP. However, these concerns appear to be unwarranted for women who recall their LMP to be within 56 days, with a degree of certainty. Recognizing this, the recent guidelines for medical abortion (Canada) state that ultrasound is not needed to determine gestational age eligibility where women are sure of their LMP [24].

Recall is usually vaguer over longer periods of time and discrepancies between LMP based GA and U/S GA increase at later gestational ages [5, 9, 12, 18]. In addition, a higher likelihood of underestimating gestational age among women seeking abortion compared to women planning to take the pregnancy to term has been reported [12]. However, in this study CHWs did not often evaluate women in the second trimester as eligible for early medical abortion – most women with advanced pregnancies were likely to be correctly identified by CHWs as requiring referral to centers providing second trimester services.

In South Africa and in many other middle-income countries, medical abortion up to 63 days gestational age is provided at broad-based primary care level, while vacuum aspiration is done for gestations up to 84 days, but is often only available at urban hospitals. Thus screening and referral by CHWs, either community or facility-based, has potential to save women’s time and reduce delays by referring her directly to an appropriate facility that will provide her with an abortion method of her choice and at first visit. As LMP for gestational age estimation is self-reported information, it may become commonplace for women to use an online pregnancy calculator based on LMP to self-assess whether they are eligible for medical abortion. As such, our findings contribute to the growing body of evidence supporting novel service delivery models in which women are able to safely use medical abortion without the involvement of a healthcare intermediary. Self-management or task sharing of gestational age assessment could relieve pressure from providers, and pave the way for increased access to medical abortion particularly in under-resourced facilities where U/S is not available. For women not eligible or not wanting a medical abortion, providers skilled in bimanual pelvic examination can safely determine gestational age eligibility for first trimester abortions involving vacuum aspiration.

Study limitations included limited generalizability due to the selection of NGO health services for the study and that CHWs received specific training. We did not ask women whether they had regular menstrual cycles, which might be a significant factor determining accurate recall of LMP. Further investigation into women’s perspectives on their own assessment of gestational age, or how they feel about task sharing this component of care with other cadres of healthcare providers is warranted. Sustained and broad-based implementation of task sharing would require careful planning and should meet women’s privacy needs, address health-care workers’ concerns on managing procedural risk and include training, support and certification for CHWs. In addition, the concerns of providers with respect to their clinical domain need to be addressed during this process.

## Conclusions

In South Africa, if gestational age calculated from LMP is within 56 days, this would ensure a safe and effective medical abortion is provided in most cases. If unsure of their LMP, and for those with further advanced pregnancies, women would be advised to seek more specialized services. If signs of a failed procedure or excessive bleeding are explained to women and additional care is readily available if needed, task sharing or self-assessment of gestational age from LMP could expand access and strengthen medical abortion services at primary care level.

## Abbreviations

CHW: Community health worker
GA: Gestational age
IDI: In-depth interview
LMP: Last menstrual period
MA: Medical abortion
NGO: Non-governmental organization
SD: Standard deviation
U/S: Ultrasound
US: United States
ZAR: South African Rands (currency)
